# Separation and Washing of Candida Cells from White Blood Cells Using Viscoelastic Microfluidics

**DOI:** 10.3390/mi14040712

**Published:** 2023-03-23

**Authors:** Hyunjung Lim, Jae Young Kim, Seunghee Choo, Changseok Lee, Byoung Joe Han, Chae Seung Lim, Jeonghun Nam

**Affiliations:** 1 School of Biomedical Engineering, Korea University, Seoul 02841, Republic of Korea; 2 Research Institute for Skin Image, Korea University College of Medicine, Seoul 08308, Republic of Korea; 3 Core Research & Development Center, Korea University Ansan Hospital, Ansan 15355, Republic of Korea; 4 College of Life Sciences and Bio Engineering, Incheon National University, Incheon 22012, Republic of Korea; 5 Department of AI Electrical and Electronic Engineering, Incheon Jaeneung University, Incheon 22573, Republic of Korea; 6 Department of Laboratory Medicine, College of Medicine, Korea University, Seoul 08307, Republic of Korea; 7 Artificial Intelligence(AI)-Bio Research Center, Incheon Jaeneung University, Incheon 21987, Republic of Korea

**Keywords:** Candida, white blood cell, separation, washing, viscoelastic fluid

## Abstract

An early and accurate diagnosis of Candida albicans is critical for the rapid antifungal treatment of candidemia, a mortal bloodstream infection. This study demonstrates viscoelastic microfluidic techniques for continuous separation, concentration, and subsequent washing of Candida cells in the blood. The total sample preparation system contains two-step microfluidic devices: a closed-loop separation and concentration device and a co-flow cell-washing device. To determine the flow conditions of the closed-loop device, such as the flow rate factor, a mixture of 4 and 13 μm particles was used. Candida cells were successfully separated from the white blood cells (WBCs) and concentrated by 74.6-fold in the sample reservoir of the closed-loop system at 800 μL/min with a flow rate factor of 3.3. In addition, the collected Candida cells were washed with washing buffer (deionized water) in the microchannels with an aspect ratio of 2 at a total flow rate of 100 μL/min. Finally, Candida cells at extremely low concentrations (Ct > 35) became detectable after the removal of WBCs, the additional buffer solution in the closed-loop system (Ct = 30.3 ± 1.3), and further removal of blood lysate and washing (Ct = 23.3 ± 1.6).

## 1. Introduction

Candidemia is a bloodstream infection caused by Candida and is the most common form of candidiasis [1]. Among more than 150 species of the genus Candida, *Candida albicans* (*C. albicans*) is the most pathogenic type and has a 40–54% mortality rate in hospitalized patients [2]. Therefore, early diagnosis of *C. albicans* is essential for the rapid antifungal treatment of fungal infections [3]. The conventional method for the diagnosis of Candida fungal infections is a blood culture [4], though it is critically limited because it can overlook extremely low concentrations of Candida in the blood and has a long duration. To address the current limitations, a genomic amplification method, the real-time polymerase chain reaction (RT-PCR), has been used as a non-culture-based diagnostic method with high sensitivity (~95%) and specificity (~92%) [5]. However, the sensitivity of PCR can be compromised by other nucleated cellular products such as white blood cells (WBCs), blood cell lysates, and low concentrations of Candida cells. Multi-step centrifugation processes are still required to enhance PCR analysis results [6]. Therefore, it is necessary to develop methods for the separation, concentration, and washing of Candida cells.

Recent advancements in microfluidic technology have enabled the increased use of microfluidic-based separation and concentration of particles/cells in biological and clinical applications [7,8]. Microfluidic techniques for separation and concentration can be divided into two categories depending on the use of external force fields (active and passive techniques). Active techniques adopt various force fields, including electric [9,10], magnetic [11,12], optical [13], and acoustic forces [14,15], whereas passive techniques utilize the channel geometry and/or hydrodynamic effects of the flow without using external forces [16].

Cell washing is necessary for sample preparation before biological and clinical analyses [17,18] because a medium exchange of cells from a high background to a washing buffer at a low background can enhance the sensitivity and accuracy of the analysis results. The standard method for cell washing is centrifugation, which is limited by possible cell damage induced by high shear conditions, manual pipetting steps, and batch processes [19]. Therefore, microfluidic technique-based cell washing has been widely used as an alternative to centrifugation. These microfluidic cell-washing techniques include active and passive methods [19], which are all conducted in Newtonian fluids.

Recently, viscoelastic non-Newtonian microfluidics has gained much attention based on intrinsic nonlinear elastic forces in pressure-driven flows [20]. Compared to other passive methods using Newtonian fluids, viscoelastic particle/cell manipulation can be achieved in a simple straight microchannel due to the non-uniform distribution of the first normal stress difference (*N*_1_) [21], eliminating the need for complicated channel structures. Therefore, viscoelastic microfluidics has been applied to the focusing [21,22], separation [23,24,25,26,27], concentration [28,29], and washing of particles/cells [19].

In this study, we propose two-step viscoelastic microfluidic devices to achieve closed-loop separation and concentration, followed by continuous cell washing of *C. albicans*. To the best of the authors’ knowledge, microfluidic techniques have not been applied to the continuous separation, concentration, and washing of fungi in blood for high-sensitivity molecular detection. We evaluated the device’s performance using particles of different sizes depending on the suction flow rates to optimize the flow conditions of the closed-loop separation system. In addition, the washing performance of our device was examined by measuring the absorbance of the collected samples depending on the sample-to-sheath flow rate ratios. Finally, our devices were used to separate, concentrate, and wash Candida cells for clinical diagnosis. The device’s performance was validated by post-analysis using RT-PCR.

## 2. Materials and Methods

### 2.1. Device Fabrication

Two-step microfluidic devices were used for viscoelastic closed-loop separation, concentration, and co-flow washing. The first-step closed-loop device consisted of four parallel microchannels with one inlet and two outlets. The channel comprised the first stage for cell focusing at the centerline and the second stage for size-based cell separation [24]. The device has a high aspect ratio (α=h/w, where *h* is the height and *w* is the width) cross-section with a width of 40 μm in the 1st stage and 70 μm in the 2nd stage and a height of 125 μm. The 1st stage width was determined to achieve a blockage ratio (β=a/w, where *a* is the particle/cell diameter) larger than 0.1 for Candida, which is known to be approximately 4 μm in diameter (β=0.1). The second-step co-flow device consisted of two inlets and two outlets. The width and height of the channel were 40 and 80 μm, respectively, with an aspect ratio of 2. The exact device design and channel size were given in Figure S1 in the Supplementary Information.

A polydimethylsiloxane (PDMS) microchannel was fabricated using a conventional soft lithography technique. The channel was fabricated on a replica mold using an SU-8 negative photoresist (MicroChem, Newton, MA, USA) on a silicon wafer. The PDMS base and curing agent (Sylgard 184, Dow Corning, Midland, MI, USA) were mixed using a 10:1 ratio, degassed in a vacuum chamber, and thermally cured in an oven for 1 h at 80 °C. The cured PDMS channels were peeled off and bonded to a glass slide using oxygen plasma (Femto Science, Hwaseong, Republic of Korea).

### 2.2. Sample Preparation

A viscoelastic non-Newtonian fluid, 0.1% (*w*/*v*) hyaluronic acid (HA) sodium salt (357 kDa, Lifecore Biomedical, Chaska, MN, USA), was prepared in phosphate-buffered saline. The high shear viscosity and relaxation time of the solution were 0.89 mPa∙s and 0.25 ms, respectively [30]. Fluorescent polystyrene particles with diameters of 4 (green, ThermoFisher, Waltham, MA, USA) and 13 μm (red, ThermoFisher, Waltham, MA, USA) were used to examine the flow characteristics prior to application to the biological samples. The particle diameters were chosen to serve as analogs to Candida cells and WBCs, respectively. The particles were suspended in a 0.1% HA solution at approximately 1 × 10^5^ particles/mL.

Single-donor human whole blood (Innovative Research Inc., Novi, MI, USA) was used in this study. *C. albicans* SC5413 was provided by Dr. Jeong-Yoon Kim at the Department of Microbiology and Molecular Biology, Chungnam University, Republic of Korea. Yeasts were cultured overnight at 30 °C in 10 mL of yeast extract-peptone-dextrose (YPD) broth (Qbiogene, Inc., Carlsbad, CA, USA), and the cultured cells were quantified by phase-contrast microscopy (40× power) using a counting grid. For the final biological application, 1 mL of whole blood was mixed with 8 mL 1 × BD FACS lysing solution (BD Biosciences, San Jose, CA, USA) and 1 mL of 1% HA solution containing 100 nm fluorescent particles (R100, ThermoFisher, Waltham, MA, USA). Therefore, the final concentration of the HA solution was 0.1%. Nano-sized fluorescent particles were added to visualize the viscoelastic fluid flow in the second-step co-flow system. The final concentrations of WBCs and *C. albicans* were approximately $2\times 10^{5}$ cells/mL and $6\times 10^{3}$ cells/mL, respectively. To examine the possibility of clinical applications of our microfluidic system, we determined that the concentration ratio of Candida cells to WBCs was relatively high compared to the early stages of clinical cases [31,32,33].

### 2.3. Experimental Procedure and Post-Analysis

The injection and suction flow in the first-step closed-loop device were controlled by a peristaltic pump (Reglo ICC, Ismatec, Wertheim, Germany), whereas the sample and sheath fluids were injected into the second-step co-flow device using a syringe pump (Fusion-4000, Chemyx, Stafford, TX, USA). During the experiment, the particles and cells were monitored using an inverted microscope (IX71, Olympus, Tokyo, Japan) equipped with a color CCD camera (CS230B, Olympus, Tokyo, Japan). The numbers of Candida cells and WBCs were counted manually using a hemocytometer.

To validate device performance, real-time (RT) PCR was performed using a CFX-96 instrument (BioRad, Hercules, CA, USA). DNA was extracted from whole blood as described previously [34], with minor modifications. Red blood cells were briefly lysed in red cell lysis buffer (Invitrogen, Waltham, MA, USA) for 10 min at 37 °C. After centrifugation at 3000 rpm for 10 min, the pellets were treated with 200 μL of 1 M sorbitol and 5 U/μL of zymolyase (Invitrogen, Waltham, MA, USA) at 37 °C for 30 min. DNA from each sample was extracted using the QIAmp DNA Mini Kit (Qiagen, Hilden, Germany) following the manufacturer’s instructions. RT-PCR was performed using the SYBR Green Kit (Bio-Rad, Hercules, CA, USA). For *C*. *albicans* quantification, primers specific to the ITS1-ITS2 region of *C. albicans* were used (forward, TTTATCAACTTGTCACACCAGA and reverse, ATCCCGCCTTACCACTACCG65). The qPCR reaction mixture contained 2 μL of gDNA, 10 μL of iQ SYBR Green Supermix (2×), 1 μL of forward primer (5 pmol/μL), 1 μL of reverse primer (5 pmol/μL), and RNase-free water in a total volume of 20 μL. The cycling conditions were as follows: initial denaturation at 94 °C for 3 min, followed by 35 cycles of denaturation at 95 °C for 20 s, and annealing and elongation at 60 °C for 40 s.

## 3. Results

**Figure 1:**
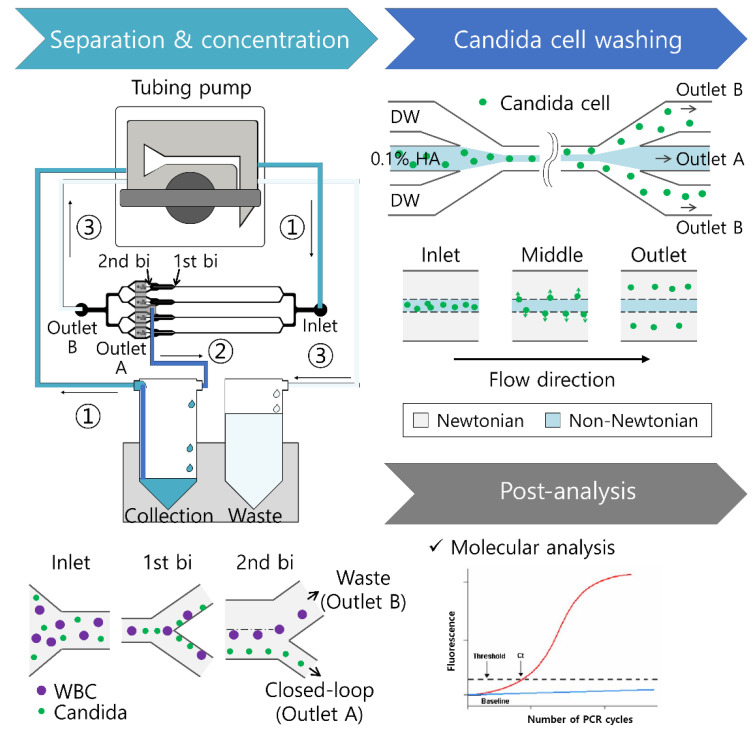
Schematic of continuous separation, concentration, and purification of candida cells using viscoelastic fluid.

A schematic representation of the workflow and microfluidic devices used for continuous separation, concentration, and washing are shown in Figure 1. The workflow consisted of three steps: (1) a closed-loop system for cell separation and concentration; (2) Candida cell washing; and (3) post-analysis using RT-PCR. The first-step device was a closed-loop system that used a microfluidic device with four parallel channels to reduce the flow resistance of the microchannel for closed-loop operation. As shown in Figure 1, randomly distributed cells were injected into the microchannel using a tubing pump (process ① in Figure 1), and all cells were focused along the centerline under the viscoelastic effect for initialization at the 1st bifurcation (1st bi) of each channel among four parallel channels prior to separation. Then, size-based cell separation was achieved due to a size-dependent elastic force ($F_{e}\sim a^{3}$, where *a* is the particle/cell diameter) at the 2nd bifurcation (2nd bi) of each microchannel [35,36]. The detailed working principle of each channel of the first-step device has been described elsewhere [24]. Separated Candida cells at the center outlet were recirculated to the microchannel (process ② in Figure 1), whereas WBCs were continuously removed from the side outlet (outlet B of the first-step device) (process ③ in Figure 1). Therefore, Candida cells were highly concentrated at the center outlets and were collected from the sample reservoir. The center outlets of four parallel channels were connected to a single outlet A of the first-step device. Then, as shown in “Candida cell washing” in Figure 1, concentrated Candida cells were injected into the center inlet of the second-step single co-flow device. Deionized water (DW) was used as the sheath fluid at the rear inlet. Candida cells and the blood lysates were initially suspended in a 0.1% HA solution. During the flow, Candida cells laterally migrated across the viscoelastic/Newtonian fluid interface toward the equilibrium positions in Newtonian fluid (DW) owing to the balance of the four forces, including the elastic force (*F_e_*), inertial lift force (*F_i,L_*), wall lift force (*F_i,W_*), and Stoke’s drag force (*F_D_*) [19,37].

(1) $$F_{e}\sim a^{3}\frac{\partial N_{1}}{\partial x}\sim \lambda {\left(a/W\right)}^{3}Q^{3},$$

(2) $$F_{i}=F_{i,L}+F_{i,W}\sim \rho {\left(a/W\right)}^{4}Q^{2},$$

(3) $$F_{D}=3\pi \eta_{s}aV_{p},$$

where x, $N_{1}$, λ, *W*, *Q*, ρ, $\eta_{s}$, and $V_{p}$ are the lateral distance, first normal stress difference, relaxation time, microchannel width, flow rate, solution density, fluid viscosity, and lateral velocity of the particle/cell, respectively. Finally, Candida cells that migrated laterally to DW were washed and collected at the side outlets (outlet B of the second-step device), while the debris from lysed blood and viscoelastic non-Newtonian fluid was removed to the center outlet (outlet A of the second-step device).

Because of the simultaneous effect of various forces during the flow, nondimensional numbers, such as the Reynolds number (*Re*), Weissenberg number (*Wi*), and elasticity number (*El*), were adopted to characterize the viscoelastic flow system in microchannels. *Re* is defined as the ratio of the inertial force to the viscous force, and *Wi* is the ratio of the elastic force to the viscous force. *El* shows the relative effect of fluid elasticity on inertia, which is used to predict the significance of elasticity over inertia in particle migration dynamics.

(4) $$Re=\frac{\rho V_{m}D_{h}}{\eta },$$

(5) $$Wi=\lambda \dot{\gamma_{c}},$$

(6) $$El=\frac{Wi}{Re},$$

where $V_{m}$, $D_{h}$, η, and $\dot{\gamma_{c}}$ indicate the mean flow velocity, hydraulic diameter of the channel, characteristic viscosity of the solution, and characteristic shear rate, respectively. Finally, the washed Candida cells were analyzed by molecular analysis (RT-PCR). The baseline is the fluorescence noise level in early cycles, and the threshold is the significantly detectable increase in fluorescence, which is set before the analysis. The threshold cycle (Ct) indicates the cycle at which the amplification plot crosses the threshold value.

**Figure 2:**
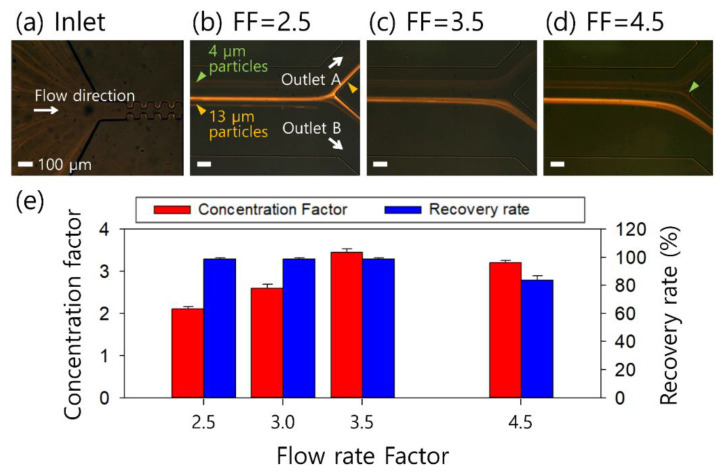
Viscoelastic separation of 4 and 13 μm particles at the (**a**) inlet and outlet depends on the flow rate factors of (**b**) 2.5, (**c**) 3.5, and (**d**) 4.5 at the inlet flow rate of 800 μL/min. Green and yellow triangles indicate 4 and 13 μm particles, respectively. (**e**) Concentration ratio and recovery rate of 4 μm particles at the center outlet (outlet A).

To determine the flow rate conditions for the separation of Candida cells and WBCs in the first-step closed-loop device, the effect of the suction flow rates at the rear outlet (outlet B) on the flow characteristics of 4 and 13 μm particles was examined. Figure 2 shows the viscoelastic separation of 4 and 13 μm particles depending on the flow rate factor, which is the ratio of the inlet flow rate to the outlet flow rate at the center outlet (outlet A) of the outlet region [31]. At the inlet of the microchannel, a mixture of 4 and 13 μm particles was randomly injected at a total flow rate of 800 μL/min (Figure 2a). At the outlet bifurcation, 13 μm particles (β=13/70=0.18>0.1) migrated laterally to the center of the 70 μm-width channel, compared to 4 μm particles (β=4/70=0.05<0.1). In this study, the widths of the outlet channels were designed to be 200 μm at outlet A and 300 μm at outlet B, such that the initial flow rate factor at outlet A was determined to be 2.5. To further manipulate the flow rate factor to optimize device performance, the suction flow rate at the side outlets (outlet B) was controlled from 480 to 700 μL/min, while the inlet flow rate was fixed at 800 μL/min. The flow-rate factor was examined, ranging from 2.5 to 8.0 at intervals of 0.5.

At FF = 2.5 (suction flow rate of 480 μL/min at outlet B), some of the laterally migrated 13 μm particles were collected with 4 μm particles at outlet A, as indicated by the yellow triangles in Figure 2b. At FF = 3.5 (suction flow rate of 572 μL/min at outlet B), 4 and 13 μm particles were separated (Figure 2c). As the suction flow rate at outlet B increased further to 622 μL/min (FF = 4.5, Figure 2d), a few 4 μm particles could not be recovered at outlet A and were deflected into outlet B, as indicated by the green triangle.

Figure 2e shows the device performance depending on the FF using the concentration factor and recovery rate. The concentration factor is defined as the ratio of the particle concentration of the sample collected at outlet A to the initial particle concentration at the inlet, whereas the recovery rate is defined as the ratio of the number of particles retrieved from outlet A to the number of particles injected at the inlet. An increase in the flow rate factor from 2.5 (suction flow rate = 480 μL/min) to 3.5 (suction flow rate = 572 μL/min) increased the concentration factor to approximately 3.5. However, as the flow rate factor increased further to FF = 4.5, the concentration factor decreased to approximately 3.2 because a certain amount of 4 μm particles flowed to outlet B. The recovery rate remained higher than 98% at a flow rate factor between 2.5 and 3.5, as shown in Figure 2e. However, at FF = 4.5, the recovery rate decreased to approximately 83.7% due to 4 μm particles deflected to outlet B. For an elaborate determination of the flow rate factor to achieve high separation efficiency, the flow rate factor ranging from 2.5 to 4.5 was examined at narrower intervals of 0.1. Therefore, the optimized flow rate factor was decided at 3.3 for the closed-loop separation and concentration.

**Figure 3:**
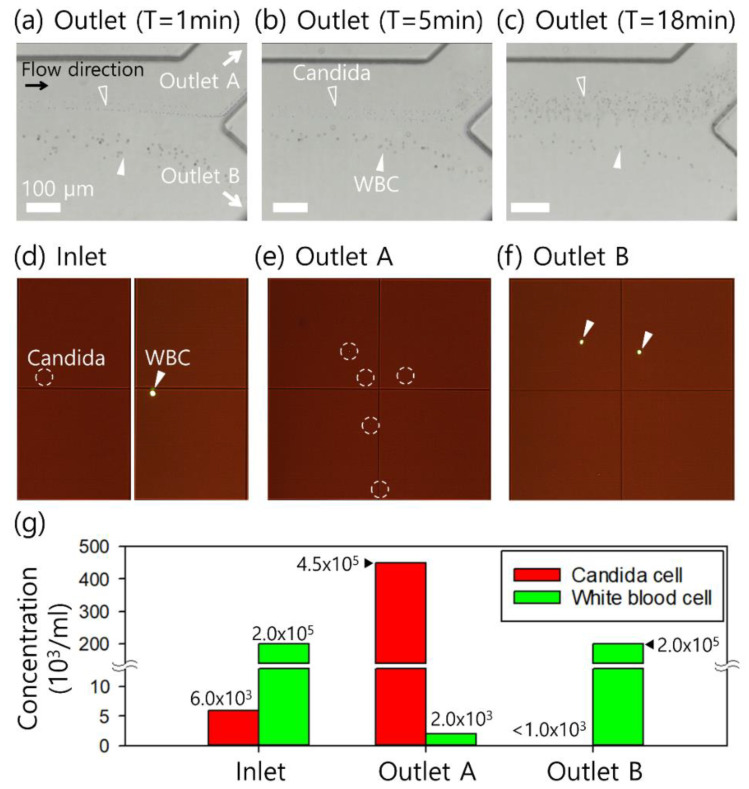
Stacked microscopic images showing viscoelastic closed-loop separation and concentration of Candida cells from white blood cells at a flow rate of 800 μL/min with FF = 3.3 at the (**a**) outlet at time T = 1 min, (**b**) outlet at time T = 5 min, and (**c**) outlet at time T = 18 min. Microscopic images of the 1:10 diluted sample before and after the closed-loop separation and concentration process at the (**d**) inlet, (**e**) outlet A, and (**f**) outlet B for manual counting. (**g**) Concentration of Candida cells and white blood cells at the inlet and outlet A and B.

To examine the performance of the closed-loop system, the time-dependent separation and concentration of Candida cells from the WBCs in a viscoelastic closed-loop system were examined. Figure 3a–c shows the stacked images of the time-dependent continuous separation and concentration of Candida cells from 10 different images captured in the single channel among the four parallel channels in the closed-loop device at an inlet flow rate of 800 μL/min with FF = 3.3 at outlet A (suction flow rate at outlet B = 558 μL/min). At the inlet, the WBCs and Candida cells were randomly distributed across the microchannels. The initial volume of the samples used in this experiment was 10 mL. After viscoelastic flow in the first-step device, Candida cells were separated from the WBCs because of the size-dependent elastic force (T = 1 min in Figure 3a). Finally, after 18 min of closed-loop system operation, the number of Candida cells flowing in the microchannel noticeably increased, while the number of WBCs in the microchannel decreased (T = 18 min in Figure 3c). After 18 min of closed-loop system operation, all samples in the sample reservoir were consumed, and the residual volume of the sample in the connecting tubing was approximately 130 μL, as the dead volume.

To evaluate the separation and concentration performance of the closed-loop device, manual counting was conducted using a hemocytometer. Figure 3d–f show microscopic images of Candida cells and WBCs before and after the separation and concentration processes. To show a clear concentration difference between Candida cells and WBCs, the samples before and after the closed-loop device system were diluted at a ratio of 1:10. WBCs were stained using CD45-FITC (eBioscience, San Diego, CA, USA), whereas cells without fluorescent staining were Candida. From the microscopic images, the concentrations of each cell at the inlet, outlet A, and outlet B were manually counted, as shown in Figure 3e. Before the separation process (inlet), the binary mixture sample contained WBCs at $2.0\times 10^{5}$ cells/mL and Candida cells at $6.0\times 10^{3}$ cells/mL. After separation, Candida was successfully separated and concentrated in the sample reservoir (outlet A), containing Candida cells at $4.5\times 10^{5}$ cells/mL and WBCs at <$2.0\times 10^{3}$ cells/mL. The results indicate that Candida cells in the final sample were concentrated approximately 74.6-fold, which is slightly lower than the expected concentration factor (~77-fold) based on the approximate residual volume of 130 μL. This might be due to the wide size distribution of Candida cells, which affects viscoelastic lateral migration. In the waste reservoir through outlet B, most WBCs were removed at $2.0\times 10^{5}$ cells/mL, and a small number of Candida cells were found to be uncountable using the manual counting method, which was lower than $1.0\times 10^{3}$ cells/mL. The purity of separated Candida cells was defined as the ratio of the number of Candida cells collected at outlet A to the total number of cells at outlet A (99.5 ± 0.2%), while the removal ratio of WBCs was defined as the ratio of the number of WBCs removed at outlet B to the total number of WBCs at both outlets A and B (99.0 ± 0.4%). As a further study for clinical optimization, the effect of Candida cell concentration on device performance, including the concentration factor and the purity of Candida cells harvested from the device, can be examined.

For a highly sensitive and accurate molecular diagnosis, Candida cells that were separated and concentrated in the first-step device were washed and collected in deionized water (DW). Conventionally, Candida cells in lysed blood require a multistep centrifugation process to remove the lysate for high-sensitivity detection [6]. In our second-step co-flow system, medium exchange was achieved for Candida cells to remove lysed blood debris and viscoelastic fluids.

**Figure 4:**
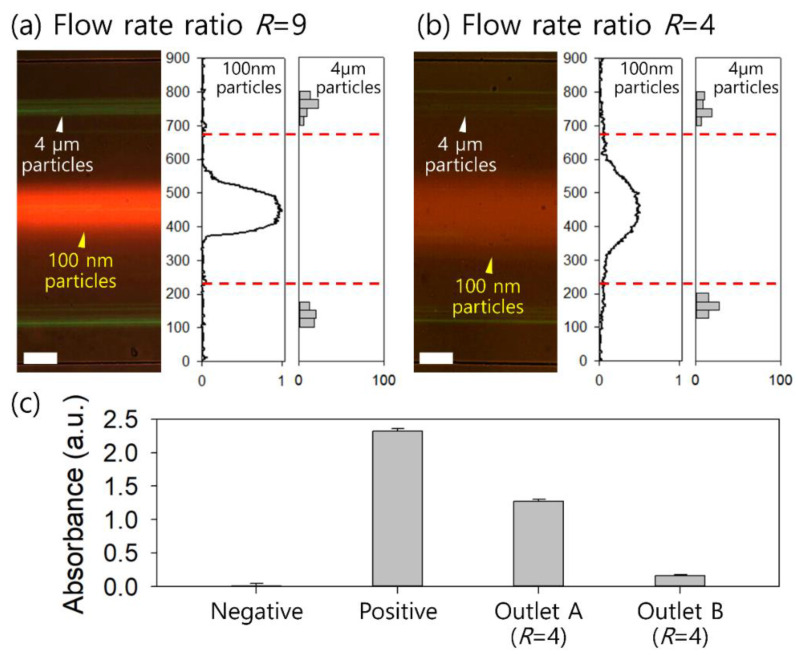
Effect of the sample-to-sheath flow rate ratio (*R*) of (**a**) 9 and (**b**) 4 on the medium exchange of 4 μm particles from a 0.1% HA solution to deionized water (DW) in the co-flow device with AR = 2. The total flow rates were 100 μL/min. (**c**) Absorbances measured by the UV-VIS spectrophotometer at 254 nm wavelength using the negative sample (inlet A), the positive sample (inlet B), and collected samples at the outlets with a flow rate ratio of 4.

To enhance the device throughput, the effect of the sample flow rate was examined by increasing the sample flow rate in a microchannel with AR = 2. The sample-to-sheath flow rate ratio (*R*) was defined as the ratio of the sheath flow rate to the sample flow rate, which was modulated from 9 (sample 10 μL/min and sheath 90 μL/min) to 1.5 (sample 40 μL/min and sheath 60 μL/min). Figure 4a,b shows microscopic images at the 900-μm width expansion region of the microchannel and the normalized particle distribution with different flow rate ratios at a fixed inlet flow rate of 100 μL/min. With R=4 (sample 20 μL/min and sheath 80 μL/min), as shown in Figure 4b, as the ratio of the sample flow rate was increased to enhance the device throughput, the fluorescent stream of 100 nm particles became wider by approximately 1.8-fold compared to that of R=9 (Figure 4a). Based on the fluorescent distribution of the 100 nm particles in Figure 4a,b, the areas under the curves of R=9 and R=4 were compared. Considering the width of the outlet trifurcation of the microchannel (1:2:1), which was shown as the red dotted lines in Figure 4a,b, only 1.3% of the total area under the curve was located outside the boundaries with R=9, while a larger area (5%) was located outside the boundaries with R=4. As the sample-to-sheath flow rate ratio decreases, the flow distribution of 100 nm particles becomes wider, and the proportion of polymer solution flowing out of the side outlets increases. Therefore, the sample-to-sheath flow rate ratio was fixed at 4 for the final demonstration. Meanwhile, for both flow rate conditions in Figure 4a,b, most of the 4 μm particles were focused into two streams between the channel center and sidewalls. Considering the design of the outlet trifurcation of the microchannel (1:2:1), the streamline of 4 μm particles lay slightly outside of the boundaries between the center and the side outlets, and 4 μm particles flowed to the side outlets for both flow rate conditions.

To assure the washing performance with the enhanced device throughput, the absorption spectra of the samples from the inlet mixture (positive sample), sheath fluid (negative sample, DW), and two outlets were examined for the full wavelength range (200–500 nm) (see Figure S2 in the Supplementary Information). 100-nm fluorescent particles were used to visualize the viscoelastic fluid flow for the washing experiments, and these particles had absorption peaks at 254 nm. Figure 4c shows the absorbance of each sample at a fixed wavelength of 254 nm. The sample collected at outlet A with *R* = 4 showed a lower absorption intensity compared to that of the inlet sample because the ratio of the trifurcation channel was lower (1:2:1) than the flow rate ratio. The washing process reduced the absorption intensity at 254 nm by about 94% in the sample collected at outlet B with *R* = 4, which indicates that the viscoelastic fluid flowed out to outlet A. However, as the sample-to-sheath flow rate ratio decreased to 2.3 to enhance the device throughput, the absorption intensities of the collected sample at outlet B became higher. Based on the results, it is confirmed that our second-step washing system at a sample-to-sheath flow rate ratio of 4 and an inlet flow rate of 100 μL/min is capable of effectively eliminating viscoelastic polymer components and washing Candida cells with PBS for downstream analysis.

**Figure 5:**
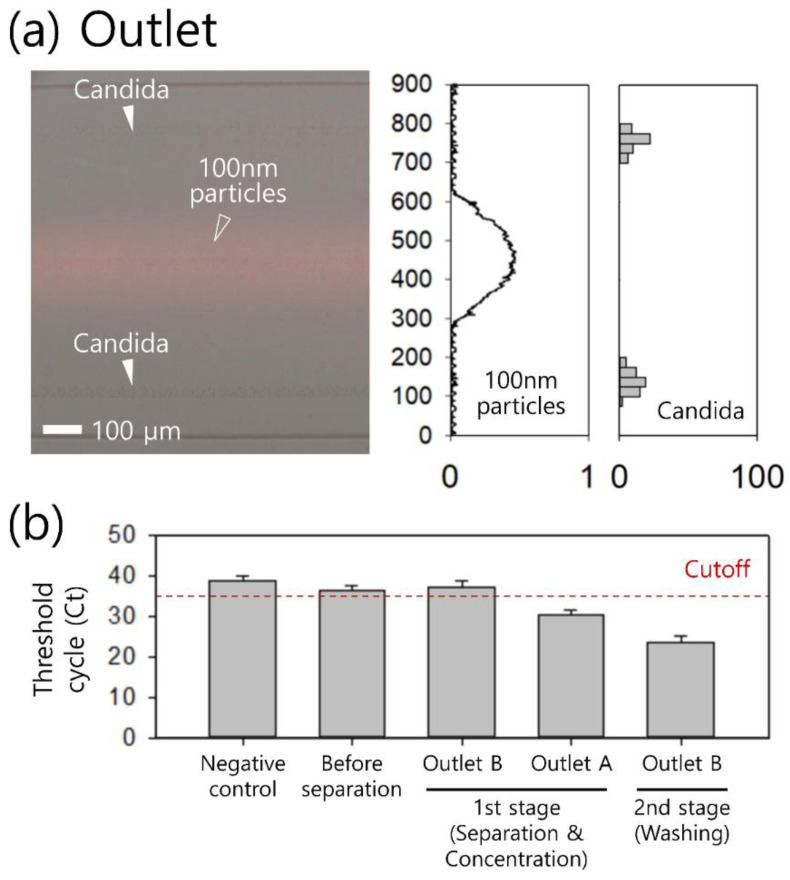
(**a**) Continuous purification of concentrated candida cells using a viscoelastic co-flow system. Stacked fluorescent microscopic images (**left**) and distribution of candida cells and 100 nm particles (**right**) at the outlet region. (**b**) The Ct values for the negative control (Ct = 38.6) represent the 0.1% (*w*/*v*) HA buffer solution. Before the separation process, candida cells could hardly be detected due to a large number of contaminants, including blood cells (Ct = 36.5). After the first-step separation, the Ct values for outlets A and B were 30.3 and 37.2, respectively. After the second-step washing process, the Ct value was measured to be 23.6. The dashed red line at Ct = 35 indicates the cutoff value for real-time PCR analysis. The error bars show the standard deviation from repeated measurements (n = 5).

For the final demonstration, Candida cells collected from the sample reservoir in the first-step closed-loop system (shown in Figure 3) were used for medium exchange. Figure 5a shows the washing of concentrated Candida cells using a viscoelastic/Newtonian co-flow device with AR = 2 at a total flow rate of 100 μL/min with a sample-to-sheath flow rate ratio of 4.

At the inlet, Candida cells suspended in a 0.1% HA solution were tightly focused along the centerline by introducing sheath fluids (DW) from side inlets. After flowing through the second-step co-flow device, Candida cells were transferred from 0.1% HA solution to DW to be washed, while the distribution of fluorescent intensity remained near the centerline. The washed Candida cells flowed from the channel sidewalls to a region within 1/4 of the channel’s width. The distribution of Candida cells was slightly wider than that of the 4 μm particles, as shown in Figure 4. This might be due to the heterogeneous size distribution of Candida cells.

To validate the device’s performance of separation, concentration, and washing of Candida cells, SYBR green RT-PCR analysis was conducted using Candida cells in blood samples at an undetectable concentration. As shown in Figure 5b, before device processing, Candida cells were barely detected due to low concentrations and contaminants in the sample, such as WBCs and blood lysate (Ct = 36.5 ± 1.0). After the separation and concentration process, the Ct values at outlets A and B of the first-step closed-loop device were 30.3 ± 1.3 and 37.2 ± 1.4, respectively. The Ct of the collected sample at outlet A decreased compared to that of the initial sample because of the simultaneous effects of the removal of WBCs and the increase in concentration. The samples collected at outlet B were not detected under PCR conditions (Ct > 35). After washing in the second-step co-flow system, the Ct value decreased further to 23.6 ± 1.6 (outlet B of the second-step washing device), due to the removal of blood lysate and viscoelastic fluid. A negative control was not detected under PCR conditions (Ct > 35).

## 4. Discussion

For the recovery of infectious microorganisms as well as candida cells, centrifugation has been generally used as the standard method. However, approximately 30 min of multi-step centrifugation and additional hands-on time for subsequent steps between centrifugation are required. Additionally, selective separation of candida cells and white blood cells cannot be achieved [6,31]. Compared to the centrifugation method, our proposed method can provide concentrated and washed candida cells within ~20 min without the need for trained personnel for additional pipetting. In addition, due to the advantages of microfluidics, the device throughput can be further improved by multiplexing the channels in parallel. In our proposed device, four channels were used in parallel for demonstration; however, the number of channels can be increased to enhance the device’s throughput. Then, the processing time of the proposed device can be reduced further. Therefore, we expect that our viscoelastic microfluidic device can be a simple but powerful tool, enabling the separation of extremely rare infectious microorganisms.

## 5. Conclusions

In summary, we have demonstrated a continuous separation, concentration, and washing method for Candida cells using a viscoelastic fluid, enabling rapid and sensitive detection of Candida cells by molecular diagnosis. The proposed method contains two-step viscoelastic microfluidic devices, including a closed-loop device for separation and concentration and a viscoelastic co-flow device for cell washing. To validate the device performance using a viscoelastic fluid, 4 and 13 μm particles were used to examine the flow characteristics depending on the flow rates. In addition, a mixture of 4 μm and 100 nm particles was used to evaluate the medium exchange over a viscoelastic/Newtonian fluid interface in microchannels at different aspect ratios with different sample-to-sheath flow rate ratios. Finally, Candida cells were successfully separated from the WBCs and concentrated by approximately 74.6-fold in the first-step device. After that, Candida cells were washed with DW to remove the blood lysate and viscoelastic polymer. Therefore, undetectable Candida cells in blood samples (Ct > 35) became detectable after using RT-PCR by sequential separation, concentration, and washing. Therefore, our devices could be widely used for sample pretreatment of extremely rare disease-related cells to improve detection sensitivity and accuracy.

## Data Availability

Not applicable.

## Supplementary Materials

The following supporting information can be downloaded at: https://www.mdpi.com/article/10.3390/mi14040712/s1, Figure S1: Computer-aided microfluidics design (CAD) of (a) the first-step closed-loop device and (b) the second-step co-flow device; Figure S2: Absorbances measured by the UV-VIS spectrophotometer at the full range of the wavelength (200–500 nm) using the negative sample (inlet A, DW), positive sample (inlet B, sample), and collected samples at the outlets with flow rate ratio of 4.

## References

[1] Pappas P.G., Kauffman C.A., Andes D.R., Clancy C.J., Marr K.A., Ostrosky-Zeichner L., Reboli A.C., Schuster M.G., Vazquez J.A., Walsh T.J. (2016). Clinical Practice Guideline for the Management of Candidiasis: 2016 Update by the Infectious Diseases Society of America. Clin. Infect. Dis..

[2] Zirkel J., Klinker H., Kuhn A., Abele-Horn M., Tappe D., Turnwald D., Einsele H., Heinz W. (2012). Epidemiology of Candida blood stream infections in patients with hematological malignancies or solid tumors. Med. Mycol..

[3] Pappas P.G., Rex J.H., Sobel J.D., Filler S.G., Dismukes W.E., Walsh T.J., Edwards J.E. (2004). Guidelines for treatment of candidiasis. Clin. Infect. Dis..

[4] Yera H., Sendid B., Francois N., Camus D., Poulain D. (2001). Contribution of serological tests and blood culture to the early diagnosis of systemic candidiasis. Eur. J. Clin. Microbiol. Infect. Dis..

[5] Avni T., Leibovici L., Paul M.J. (2011). PCR diagnosis of invasive candidiasis: Systematic review and meta-analysis. Clin. Microbiol..

[6] Flahaut M., Sanglard D., Monod M., Bille J., Rossier M.J. (1998). Rapid detection of Candida albicans in clinical samples by DNA amplification of common regions from C. albicans-secreted aspartic proteinase genes. Clin. Microbiol..

[7] Pamme N. (2007). Continuous flow separations in microfluidic devices. Lab Chip.

[8] Shields IV C.W., Reyes C.D., López G.P. (2015). Microfluidic cell sorting: A review of the advances in the separation of cells from debulking to rare cell isolation. Lab Chip.

[9] Pethig R. (2010). Review article-dielectrophoresis: Status of the theory, technology, and applications. Biomicrofluidics.

[10] Gascoyne P.R., Noshari J., Anderson T.J., Becker F.F. (2009). Isolation of rare cells from cell mixtures by dielectrophoresis. Electrophoresis.

[11] Hejazian M., Li W., Nguyen N.-T. (2015). Lab on a chip for continuous-flow magnetic cell separation. Lab Chip.

[12] Zhao W., Cheng R., Miller J.R., Mao L. (2016). Label-Free Microfluidic Manipulation of Particles and Cells in Magnetic Liquids. Adv. Func. Mat..

[13] Kayani A.A., Khoshmanesh K., Ward S.A., Mitchell A., Kalantar-Zadeh K. (2012). Optofluidics incorporating actively controlled micro- and nano-particles. Biomicrofluidics.

[14] Laurell T., Petersson F., Nilsson A. (2007). Chip integrated strategies for acoustic separation and manipulation of cells and particles. Chem. Soc. Rev..

[15] Wang Z., Zhe J. (2011). Recent advances in particle and droplet manipulation for lab-on-a-chip devices based on surface acoustic waves. Lab Chip.

[16] Martel J.M., Toner M. (2014). Inertial Focusing in Microfluidics. Annu. Rev. Biomed. Eng..

[17] Duda D.G., Cohen K.S., Scadden D.T., Jain R.K. (2007). A protocol for phenotypic detection and enumeration of circulating endothelial cells and circulating progenitor cells in human blood. Nat. Protoc..

[18] Dineva M.A., Mahilum-Tapay L., Lee H. (2007). Sample preparation: A challenge in the development of point-of-care nucleic acid—Based assays for resource-limited settings. Analyst.

[19] Yuan D., Tan S.H., Sluyter R., Zhao Q., Yan S., Nguyen N.-T., Guo J., Zhang J., Li W. (2017). On-Chip Microparticle and Cell Washing Using Coflow of Viscoelastic Fluid and Newtonian Fluid. Anal. Chem..

[20] D’Avino G., Maffettone P., Greco F., Hulsen M.J. (2010). Viscoelasticity-induced migration of a rigid sphere in confined shear flow. Non-Newton. Fluid Mech..

[21] Leshansky A.M., Bransky A., Korin N., Dinnar U. (2007). Tunable Nonlinear Viscoelastic “Focusing” in a Microfluidic Device. Phys. Rev. Lett..

[22] Ahn S.W., Lee S.S., Lee S.J., Kim J.M. (2015). Microfluidic particle separator utilizing sheathless elasto-inertial focusing. Chem. Eng. Sci..

[23] Nam J., Lim H., Kim D., Jung H., Shin S. (2012). Continuous separation of microparticles in a microfluidic channel via the elasto-inertial effect of non-Newtonian fluid. Lab Chip.

[24] Nam J., Shin Y., Tan J.K.S., Lim Y.B., Lim C.T., Kim S. (2016). High-throughput malaria parasite separation using a viscoelastic fluid for ultrasensitive PCR detection. Lab Chip.

[25] Lu X., Zhu L., Hua R., Xuan X. (2015). Continuous sheath-free separation of particles by shape in viscoelastic fluids. Appl. Phys. Lett..

[26] Nam J., Yoon J., Jee H., Jang W.S., Lim C.S. (2020). High-Throughput Separation of Microvesicles from Whole Blood Components Using Viscoelastic Fluid. Adv. Mater. Tech..

[27] Liu P., Liu H., Yuan D., Jang D., Yan S., Li M. (2021). Separation and Enrichment of Yeast Saccharomyces cerevisiae by Shape Using Viscoelastic Microfluidics. Anal. Chem..

[28] Nam J., Jang W.S., Lim C.S. (2019). Non-electrical powered continuous cell concentration for enumeration of residual white blood cells in WBC-depleted blood using a viscoelastic fluid. Talanta.

[29] Kim J., Lim H., Jee H., Choo S., Yang M., Park S., Lee K., Park H., Lim C., Nam J. (2021). High-Throughput Cell Concentration Using A Piezoelectric Pump in Closed-Loop Viscoelastic Microfluidics. Micromachines.

[30] Lim H., Back S.M., Hwang M.H., Lee D.-H., Choi H., Nam J. (2019). Sheathless High-Throughput Circulating Tumor Cell Separation Using Viscoelastic non-Newtonian Fluid. Micromachines.

[31] Nam J., Jang W.S., Hong D.H., Lim C.S. (2019). Viscoelastic Separation and Concentration of Fungi from Blood for Highly Sensitive Molecular Diagnostics. Sci. Rep..

[32] Lu X., Chow J.J.M., Koo S.H., Tan T.Y., Jiang B., Ai Y. (2020). Enhanced Molecular Diagnosis of Bloodstream Candida Infection with Size-Based Inertial Sorting at Submicron Resolution. Anal. Chem..

[33] Iyengar S.N., Kumar T., Martensson G., Russom A. (2021). High resolution and rapid separation of bacteria from blood using elasto-inertial microfluidics. Electrophoresis.

[34] Loffler J., Hebart H., Schumacher U., Reitze H., Einsele H.J. (1997). Comparison of different methods for extraction of DNA of fungal pathogens from cultures and blood. Clin. Microbiol..

[35] Seo K.W., Byeon H.J., Huh H.K., Lee S.J. (2014). Particle migration and single-line particle focusing in microscale pipe flow of viscoelastic fluids. RSC Adv..

[36] Tehrani M.J. (1996). An Experimental-Study of Particle Migration in Pipe-Flow of Viscoelastic Fluids. J. Rheol..

[37] Lu X., Xuan X. (2015). Continuous Microfluidic Particle Separation via Elasto-Inertial Pinched Flow Fractionation. Anal. Chem..

