# Supplementary material for: Separation and Washing of Candida Cells from White Blood Cells Using Viscoelastic Microfluidics
Source: Micromachines (Basel). 2023 Mar 23;14(4):712. doi: 10.3390/mi14040712 (PMC10146744; doi:10.3390/mi14040712)
Supplement: Supplementary file 1 [file micromachines-14-00712-s001.zip › micromachines-2281709-supplementary.pdf]

Supplementary Materials

Separation and Washing of Candida Cells from White Blood Cells Using Viscoelastic Microfluidics

Hyunjung Lim [1,†], Jae Young Kim [2,3,†], Seunghee Choo [4], Changseok Lee [5], Byoung Joe Han [5], Chae Seung Lim [6,*], Jeonghun Nam [5,7,*]

[1] School of Biomedical Engineering, Korea University, Seoul 02841, Republic of Korea;
[2] Research Institute for Skin Image, Korea University College of Medicine, Seoul 08308, Republic of Korea
[3] Core Research & Development Center, Korea University Ansan Hospital, Ansan 15355, Republic of Korea
[4] College of Life Sciences and Bio Engineering, Incheon National University, Incheon 22012, Republic of Korea
[5] Department of AI Electrical and Electronic Engineering, Incheon Jaeneung University, Incheon 22573, Republic of Korea
[6] Department of Laboratory Medicine, College of Medicine, Korea University, Seoul 08307, Republic of Korea
[7] Artificial Intelligence(AI)-Bio Research Center, Incheon Jaeneung University, Incheon 21987, Republic of Korea

* Correspondence: malarim@korea.ac.kr (C.S.L.); jhnam77@gmail.com (J.N.); Tel.: +82-2-2626-1514 (C.S.L.); +82-32-890-7515 (J.N.)
† These authors contributed equally to this work.

Design of two-step microfluidic devices

Two-step microfluidic devices were used for viscoelastic closed-loop separation, concentration, and co-flow washing. Figure S1 shows the exact device design and channel size of the first-step closed-loop device and the second-step co-flow device.

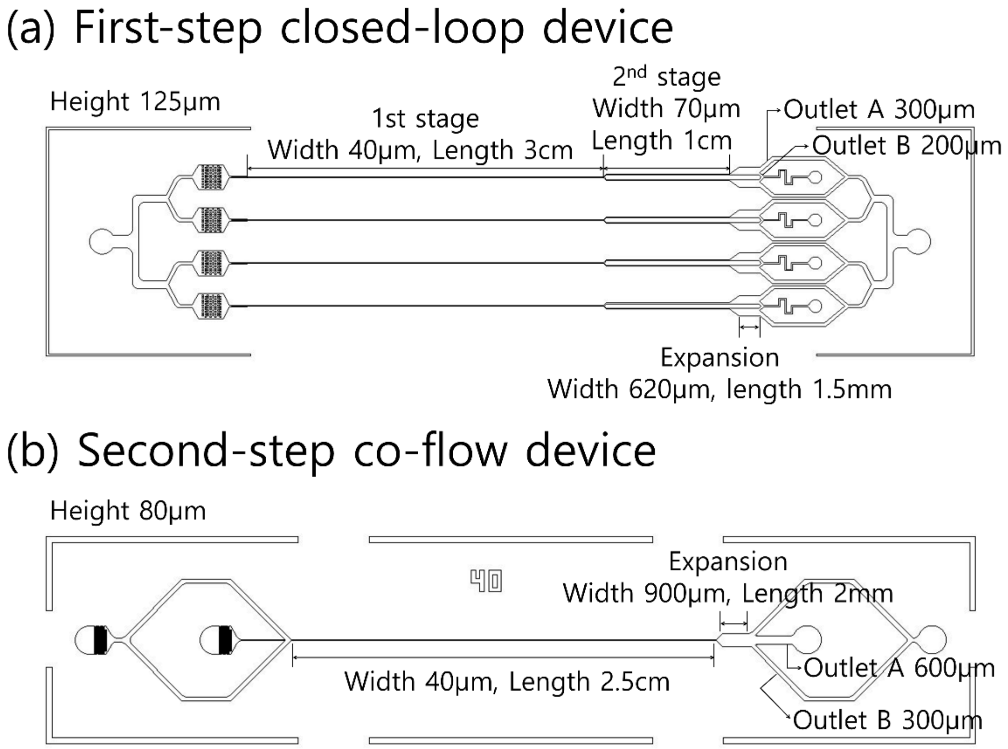

Figure S1. Computer-aided microfluidics design (CAD) of (a) the first-step closed-loop device and (b) the second-step co-flow device.

Absorption spectra of the samples

To evaluate the washing performance, the absorption spectra of the samples from the inlet mixture (positive sample), the sheath fluid (negative sample, DW), and two outlets were examined for the full range of the wavelength (200-500 nm). For visualization of the viscoelastic fluid, 100-nm fluorescent particles were used.

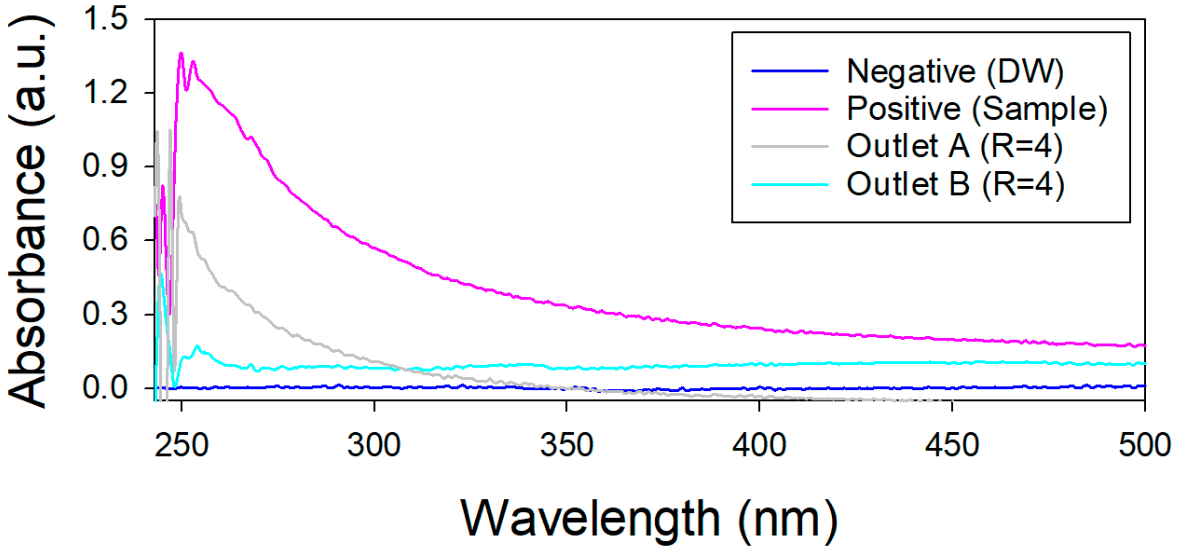

Figure S2. Absorbances measured by the UV-VIS spectrophotometer at the full range of the wavelength (200-500 nm) using the negative sample (inlet A, DW), positive sample (inlet B, sample), and collected samples at the outlets with flow rate ratio of 4.
